# Supplementary material for: Effects of microtubule (de)tyrosination on the morphology and motility of Trypanosoma brucei and cross-talk with polyglutamylation
Source: Biol Open. 2025 Dec 16;14(12):bio062270. doi: 10.1242/bio.062270 (PMC12714142; doi:10.1242/bio.062270)
Supplement: Supplementary information [file biolopen-14-062270-s1.pdf]

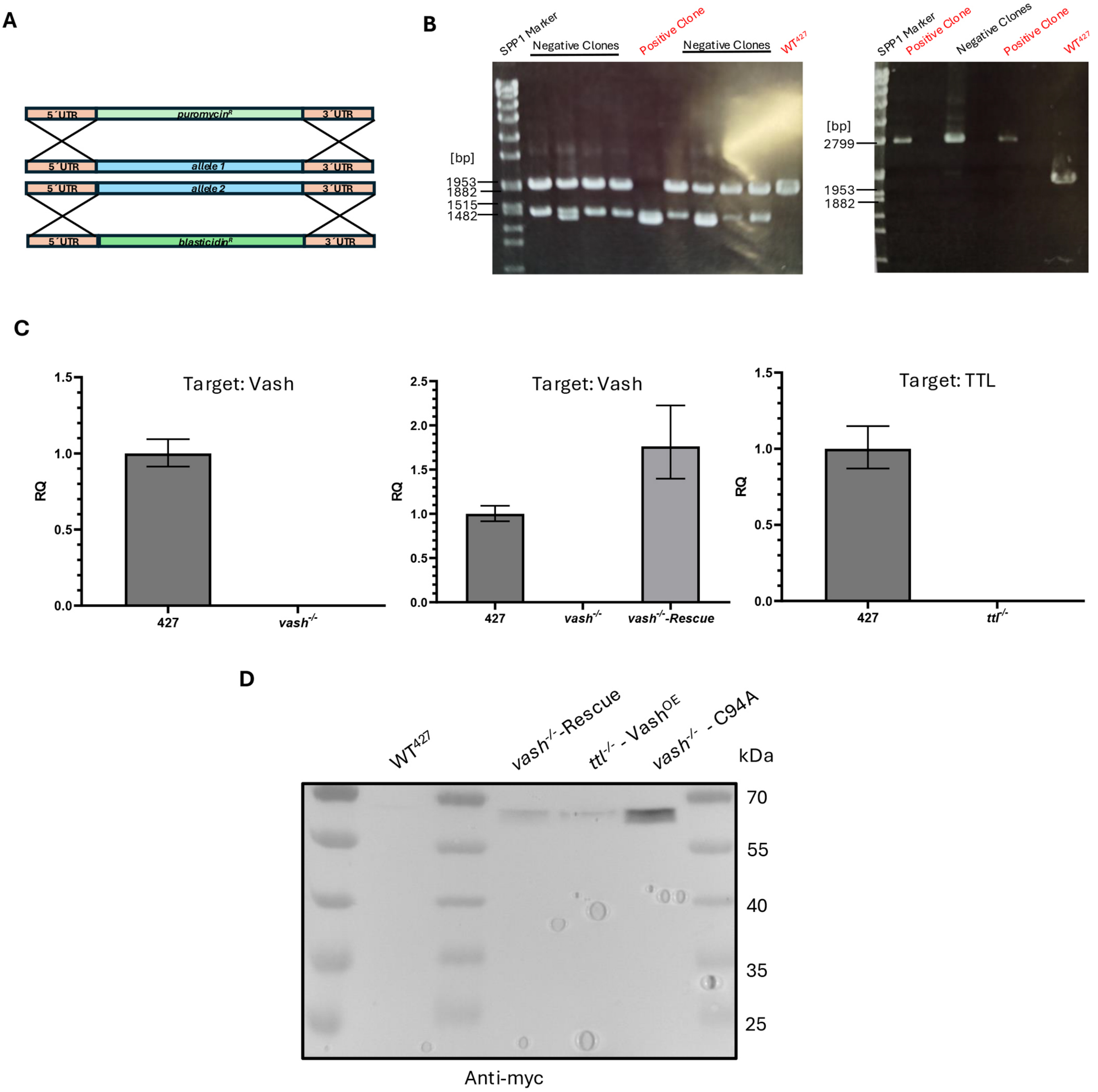

Fig. S1. Gene knockout strategy, RT-qPCR analysis and verification of correct protein expression.

A) Schematic representation of the construction of the double knockouts of either the vash and *ttl gene*. Both alleles are exchanged with puromycin and blasticidin resistance genes through homologous recombination with the respective 5´and 3´UTRs. **B)** Analysis of the correct insertion of the knockout constructs via PCR of isolated genomic DNA. The DNA in the PCR was separated using a 1% agarose gel stained with ethidium bromide. Positive clones showed a distinct band at 1349 and 1226bp for the

Biology Open • Supplementary information

VASH knockout and at 2911 and 2835bp for the *TTL* knockout, while they lack the wilde type allele band at 1979 (*VASH* knockout) and 1877bp (*TTL* knockout). **C)** RT-qPCR analysis of mRNA levels of the *VASH* transcript in the WT^{427}, *vash*$^{-/-}$ and *vash*$^{-/-}$ - *Rescue* cells and of *TTL* transcript in the WT^{427} and *ttl*$^{-/-}$ cells. In both knockout cell lines respective transcript levels were undetectable. **D)** Western blot analysis of the successful integration of the myc-tagged gene constructs *vash-2xmyc* in the *vash*$^{-/-}$ - *Rescue* and *ttl*$^{-/-}$ - *Vash*^{OE} cells and of the myc-tagged gene construct *vash-C94A-2xmyc* in the *vash*$^{-/-}$ - *C94A* cells. The WT^{427} was used as a negative control.

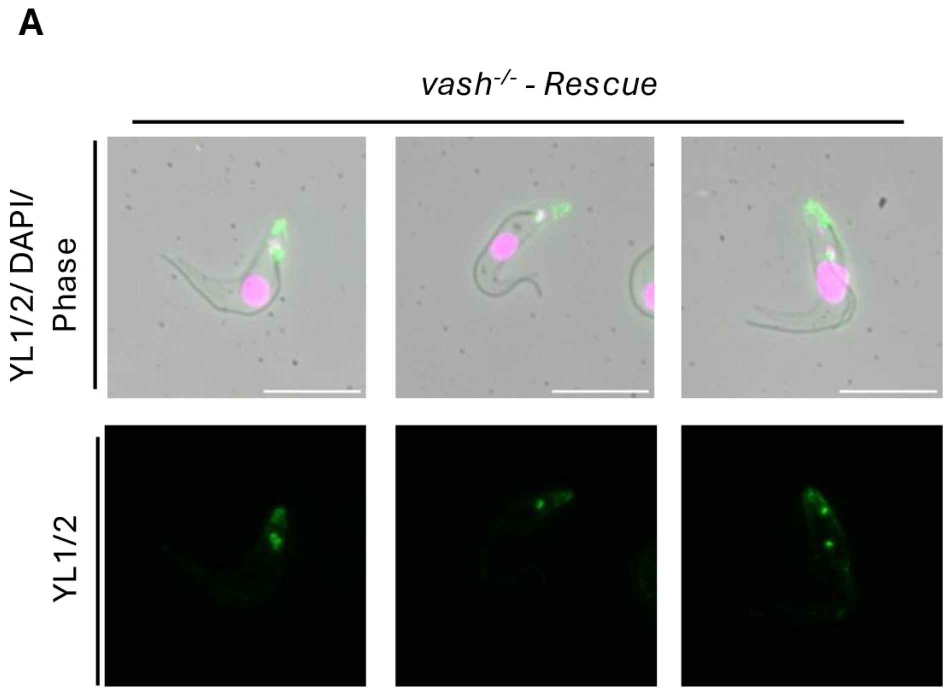

Fig. S2. The distribution of tyrosinated tubulin in the *vash*$^{-/-}$ - *Rescue* cells.

A) Immunofluorescence analysis of tyrosination levels of cytoskeletons *vash*$^{-/-}$ - *Rescue* cells. The YL1/2signal is depicted in green. The DNA was stained with DAPI (magenta). All scale bars equal 10μm.

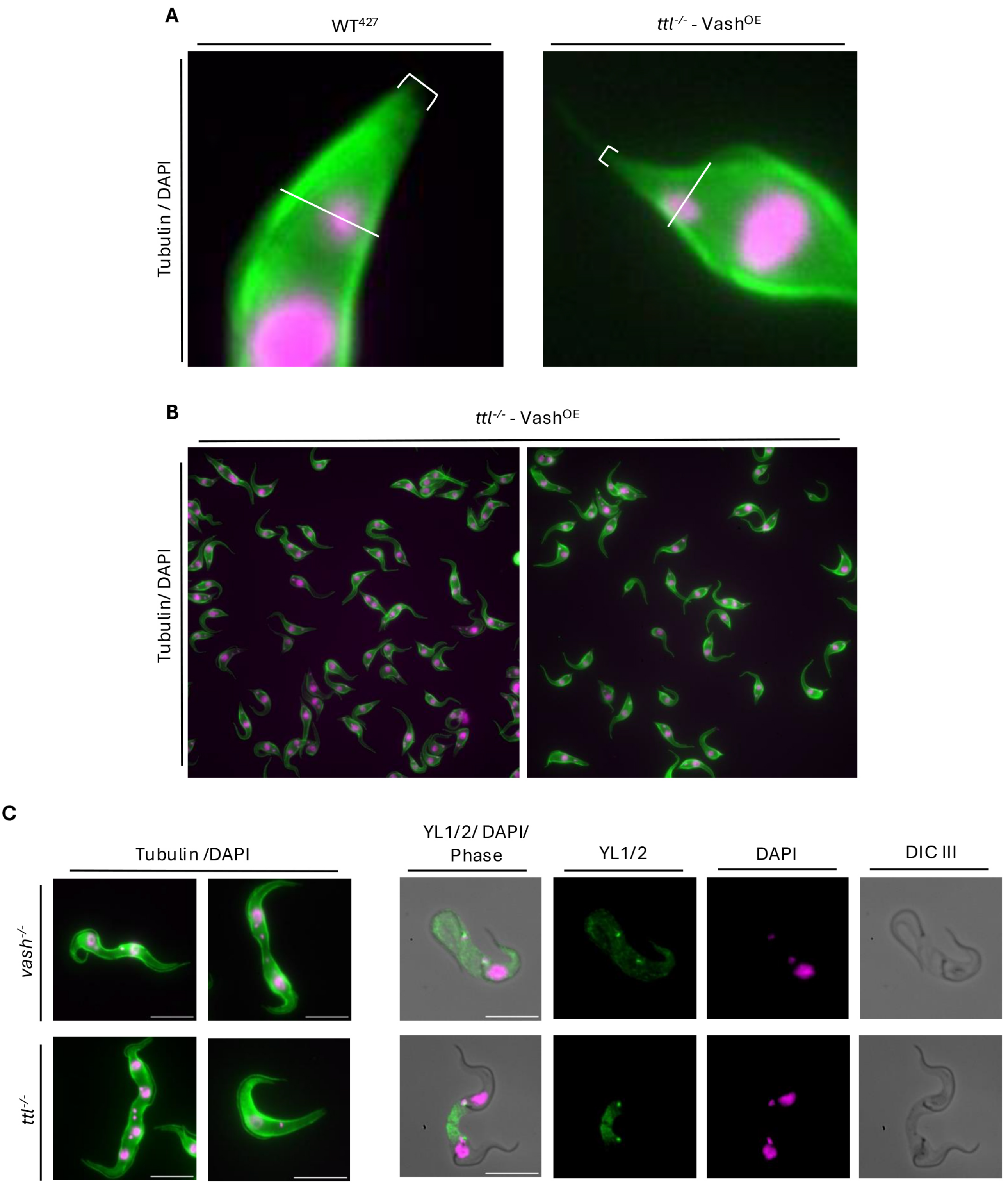

Biology Open • Supplementary information

Fig. S3. Analysis of the cellular morphology of the constructed cell lines.

A) Visualization of the different analyses of the morphology of the WT^{427} and $ttl^{-/-}$ - *Vash*^{OE} cells. The tip width of the posterior end and the cellular width at the kinetoplast was measured as depicted. The tubulin is represented in green and the DAPI signal in magenta. For better visualization of our measurement procedure, the immunofluorescence image of the WT^{427} is taken from Fig. 2A **B)** Immunofluorescence analysis of the morphology of the *ttl*$^{-/-}$ - *Vash*^{OE} cells. The tubulin signal is depicted in green and the DAPI signal in magenta. **C)** Immunofluorescence analysis of the morphology and the tyrosinated tubulin distribution in diving *ttl*$^{-/-}$ and *vash*$^{-/-}$ cells. Either the tubulin or the YL1/2 signal is depicted in green and the DAPI signal in magenta. All scale bars equal 10µm.

Biology Open • Supplementary information

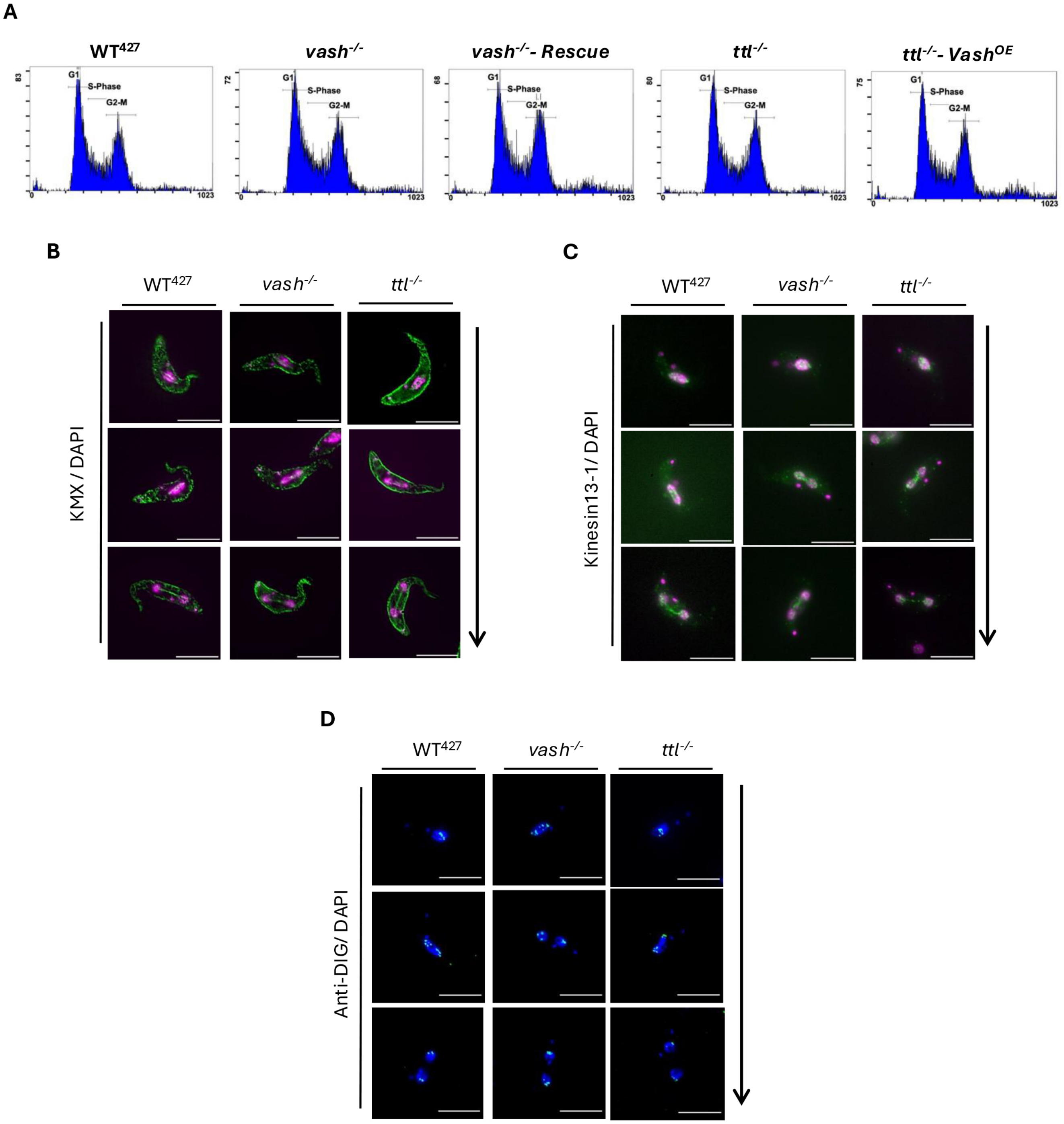

Fig. S4. Analysis of the cell cycle distribution, the formation of the mitotic spindle, Kinesin 13-1 distribution and mitotic minichromosome separation in all five cell lines. A) Flow cytometric analysis of the cell cycle distribution of WT^{427}, *vash*$^{-/-}$, *vash*$^{-/-}$ - Rescue, *ttl*$^{-/-}$ and *ttl*$^{-/-}$- *Vash*^{OE} cells**. B)** Immunofluorescence analysis of the mitotic spindle formation in WT^{427}, *vash*$^{-/-}$ and *ttl*$^{-/-}$ cells. The KMX antibody binds β-tubulin and can be used to visualize the mitotic spindle. Its signal is depicted in green and the DAPI signal in magenta. The KMX signal was deconvoluted. **C)** Immunofluorescence analysis of Kinesin 13-1 distribution during mitosis in WT^{427}, *vash*$^{-/-}$ and *ttl*$^{-/-}$ cells. The Kinesin13-1 signal is depicted in green and the DAPI signal in magenta. **D)** FISH analysis of minichromosome separation during mitosis in WT^{427}, *ttl*$^{-/-}$and *vash*$^{-/-}$ cells. Minichromosomes were *in situ* hybridized with a DIG-labelled probe, which was visualized with an anti-DIG antibody (green). The DNA was stained with DAPI (blue). The anti-DIG signal was deconvoluted. All scale bars equal 10μm. The arrows in subfigure B, C and D indicate the progress of mitosis.

Biology Open • Supplementary information

Biology Open (2025): doi:10.1242/bio.062270: Supplementary information

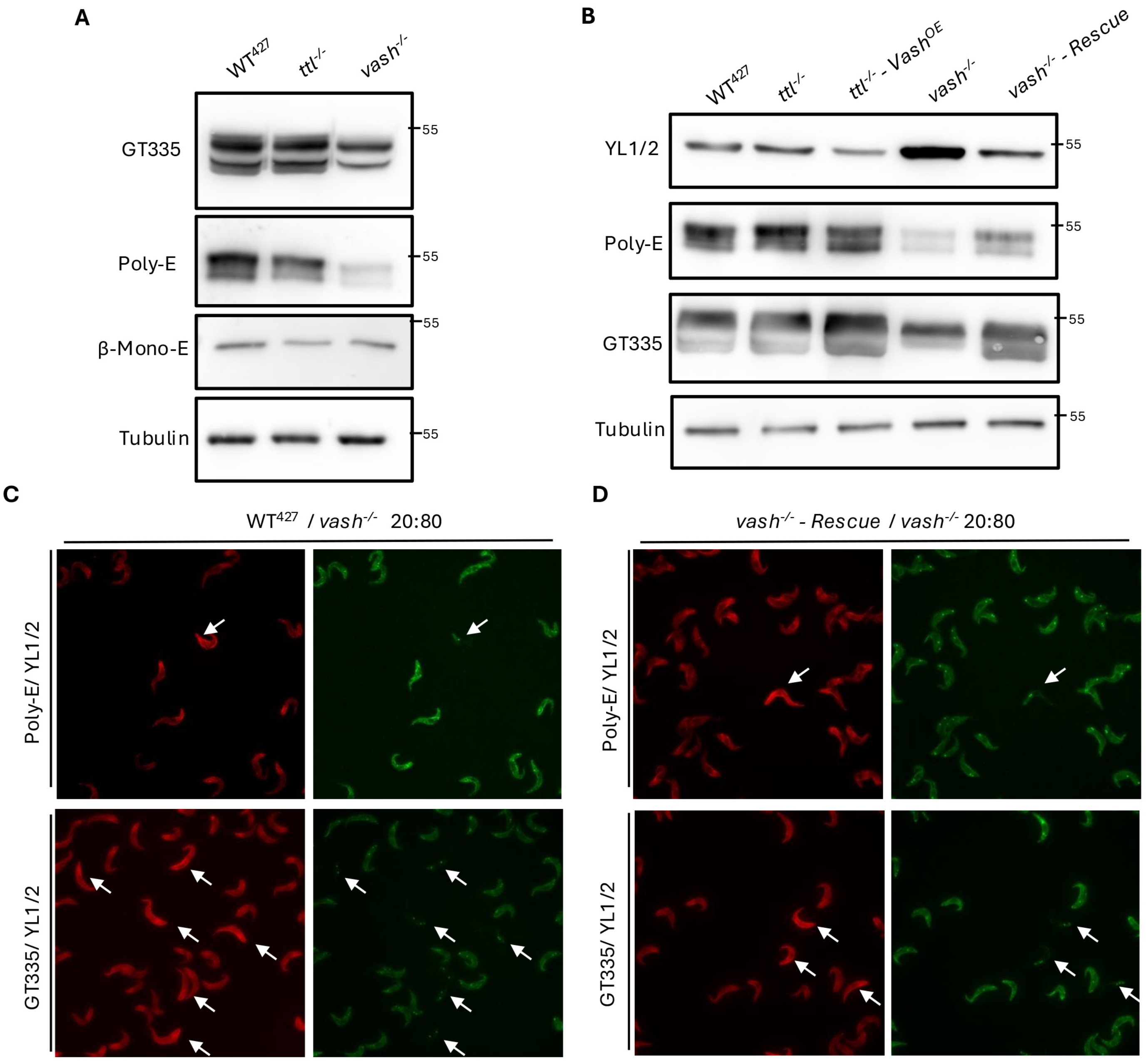

Fig. S5. Difference in polyglutamylation signal intensity in western blot analysis and in mixtures of WT^{427}, *vash$^{-/-}$* and *vash$^{-/-}$ - Rescue* cells.

A) Western blot analysis of polyglutamylation levels in cytoskeletal extracts of WT^{427}, *ttl$^{-/-}$* and *vash$^{-/-}$* cells. The β-mono-E antibody recognizes a single branching point on ß-tubulin (E435 - GE(*-E)F motif, Bodakuntla et al., 2019). The 55 kDa band of the used PageRuler Protein Ladder is indicated next to the blots as a molecular weight orientation. **B)** Western blot analysis of polyglutamylation levels in isolated flagella of WT^{427}, *ttl$^{-/-}$*, *ttl$^{-/-}$ - Vash^{OE}* , *vash$^{-/-}$* and *vash$^{-/-}$ - Rescue* cells. The 55 kDa band of the used PageRuler Protein Ladder is indicated next to the blots as a molecular weight orientation. **C)** Immunofluorescence analysis of mixtures of cytoskeletons of WT^{427} and *vash$^{-/-}$* cells. WT^{427} and *vash$^{-/-}$* cells were mixed in a 20:80 ratio and their cytoskeletons were stained with either Poly-E (red) and YL1/2 (green) or GT335 (red) and YL1/2 (green). Arrows indicate WT^{427} cells in both stainings. **D)** Immunofluorescence analysis of mixtures of cytoskeletons of *vash$^{-/-}$ - Rescue* and *vash$^{-/-}$* cells. *vash$^{-/-}$ - Rescue* and *vash$^{-/-}$* cells were mixed in a 20:80 ratio and their cytoskeletons were stained with either Poly-E (red) and YL1/2 (green) or GT335 (red) and YL1/2 (green). Arrows indicate WT^{427} cells in both stainings. All scale bars equal 10µm.

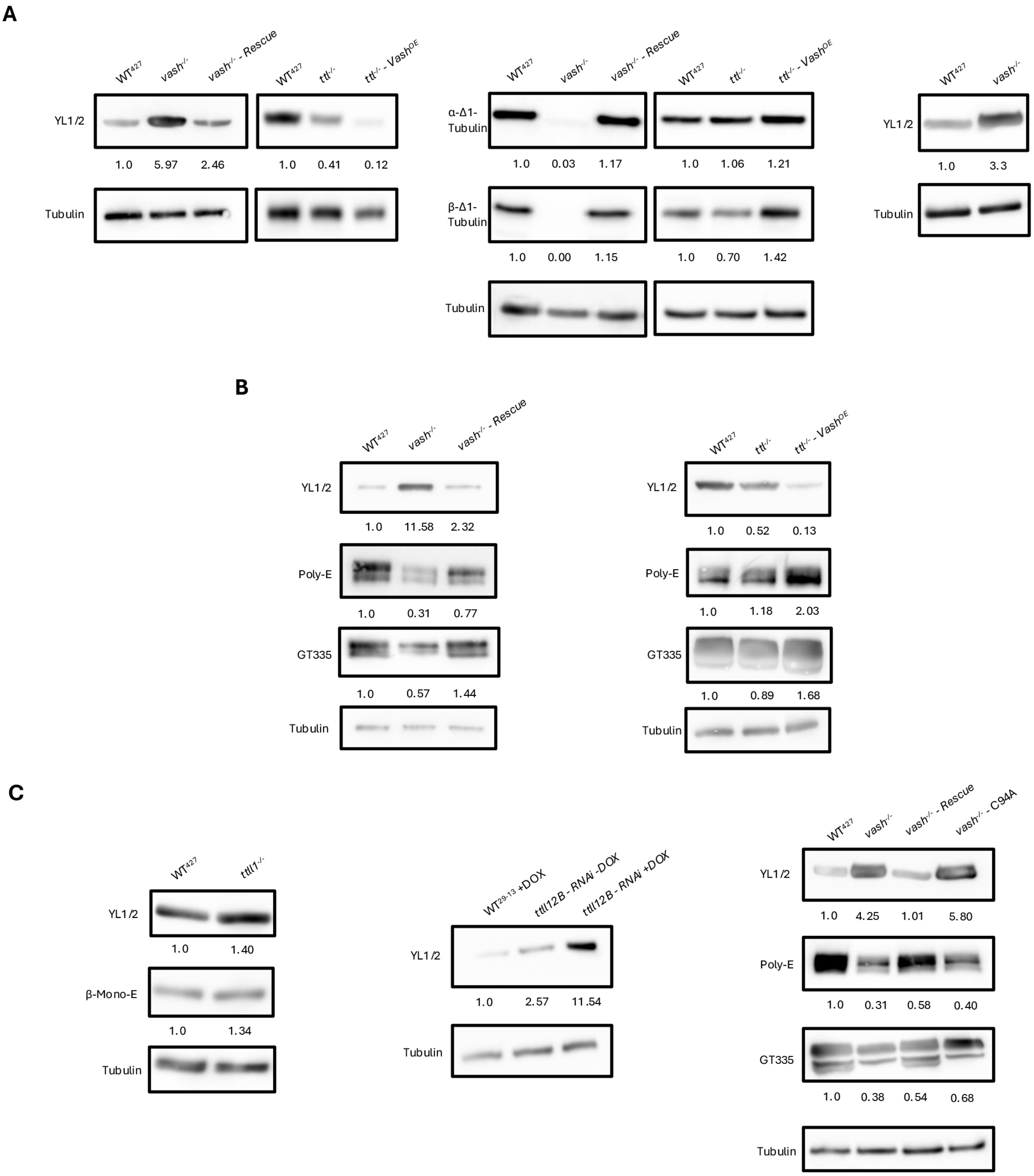

Fig. S6. Quantification of the Western Blots.

A) Quantification of the Western Blots depicted in Figure1 **B)** Quantification of the Western Blots depicted in Figure 4 **C)** Quantification of the Western Blots depicted in Figure 5. Tubulin was used as an internal loading control, and the intensities of the other blots were normalized to respective tubulin signal.

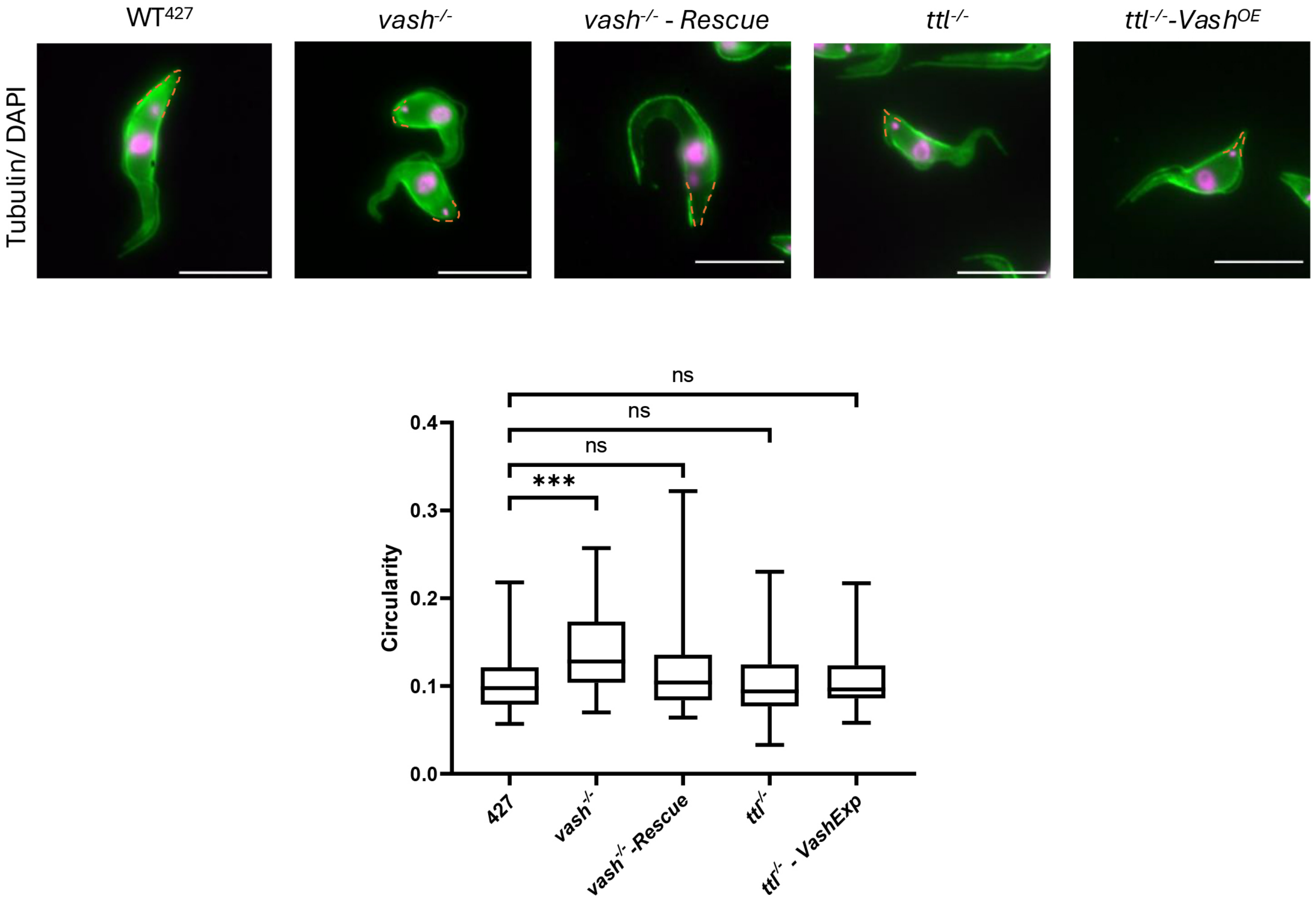

Fig. S7. Measurement of the circularity of the posterior end.

Upper panel: Schematic depiction of the measurement process with representing images of each cell line. The circularity was measured using the ImageJ function with a free hand drawn line along the edges of the tubulin immunofluorescence signal starting from the kinetoplast towards the posterior end and back. The Tubulin signal is depicted in green and the DAPI signal in magenta. For better visualization of our measurement procedure, the immunofluorescence images are taken from Fig. 2A and B and Fig. S3A respectively. Lower panel: The resulting data for the circularity of the posterior end. The boxes represent the median posterior end circularity and the interquartile range. The whiskers depict the range between the min and max values. Statistical significance is indicated above (Kruskal-Wallis test, not significant (ns) = P > 0.05, * = 0.05 > P > 0.005, ** = 0.005 > P > 0.0005, *** = 0.0005 > P, n = 50 G1-phase cells). All scale bars equals 10µm.

Table S1. Averages generation time and its standard deviation of all five cell lines.

Cell line	Average generation time [h:min ± min]
WT^{427}	9:33 ± 40
$vash^{-/-}$	9:47 ± 38
$vash^{-/-}$ - Rescue	9:46 ± 42
$ttl^{-/-}$	9:07 ± 34
$ttl^{-/-}$ - $Vash^{OE}$	8:41 ± 16

Table S2. Averages of the cell density [cells/mL] of three biological replicates, each measured three times, with their respective standard deviation.

Day	WT^{427}	*vash*$^{-/-}$	*vash*$^{-/-}$ - *Rescue*	*ttl*$^{-/-}$	*ttl*$^{-/-}$ - *Vash*^{OE}
0	$5.00·10^5$	$5.00·10^5$	$5.00·10^5$	$5.00·10^5$	$5.00·10^5$
1	$3.55·10^6$ ± $4.43·10^5$	$3.93·10^6$ ± $9.85·10^4$	$3.47·10^6$ ± $6.34·10^4$	$2.89·10^6$ ± $1.85·10^5$	$3.02·10^6$ ± $5.14·10^4$
2	$1.99·10^7$ ± $8.13·10^5$	$2.48·10^7$ ± $1.21·10^6$	$2.52·10^7$ ± $3.00·10^5$	$1.59·10^7$ ± $8.56·10^5$	$1.88·10^7$ ± $9.50·10^5$
3	$1.04·10^8$ ± $1.56·10^7$	$1.38·10^8$ ± $3.02·10^6$	$1.58·10^8$ ± $1.09·10^7$	$7.36·10^7$ ± $1.37·10^7$	$8.27·10^7$ ± $1.31·10^7$
4	$5.28·10^8$ ± $2.01·10^7$	$7.99·10^8$ ± $4.72·10^7$	$1.06·10^9$ ± $5.33·10^7$	$4.15·10^8$ ± $1.35·10^7$	$4.46·10^8$ ± $1.89·10^7$
5	$3.37·10^9$ ± $1.80·10^8$	$5.01·10^9$ ± $7.24·10^8$	$7.85·10^9$ ± $4.36·10^8$	$2.67·10^9$ ± $1.27·10^8$	$2.74·10^9$ ± $1.55·10^8$

Biology Open • Supplementary information

Table S3. List of oligonucleotides used in this study.

Application	Primer name	Sequence
TTL KO construction	TTL 5´UTR for	ATACCTGCAGGTGTTACCGCTCGTGCCAGAC
	TTL 5´UTR rev	ATATTAATTAAGGCTAACAGCGCAGACACCA
	TTL 3´UTR for	ATAGGCCGGCCCGGCACCCCGTTTCTTCCTT
	TTL 3´UTR rev	ATAGGCGCGCCTGCTGCCGCTGTAACGTGAA
Vash KO construction	Vash 5´UTR for	ATACCTGCAGGGTGCACAGCGGGTCTCTGTT
	Vash 5´UTR rev	ATATTAATTAAGTGTGGCTTCGGGTTGGTGT
	Vash 3´UTR for	ATAGGCCGGCCGTGATCACTCCCAGCGGCAA
	Vash 3´UTR rev	ATAGGCGCGCCCACAACACCCATCAGGCCGT
TTL KO analysis	TTL-KO-control for	TGTTACCGCTCGTGCCAGAC
	TTL-KO-control rev	CTCAACCTCTCCCGTGCGTC
Vash KO analysis	Vash-KO-control for	GTGCACAGCGGGTCTCTGTT
	Vash-KO-control rev	CAGCGGTAAGCTCCTGACGG
qPCR endogenous control PFR-A	qPCR-PFR-A for	CGTTGGAGATGTTTGGACCT
	qPCR-PFR-A rev	GCACGGTACTCCACCATCTT
qPCR TTL control	qPCR-TTL for	CTAACGTGGATTCTGATCGT
	qPCR-TTL rev	CATTTCCTTCAATTGCCTGG
qPCR Vash control	qPCR-Vash for	CTCGTAATACGCTGCAATTC
	qPCR-Vash rev	TATTGCCTCGTACTGCTTTT
Vash-Rescue construction	Vash-Rescue for	ATAGGCCGGCCATGTATTCTTCAGACAGGGTTCGGAC
	Vash-Rescue rev	ATAGGCGCGCCTCGATCGGCCCACATCGTTAA
Vash-C94A construction	2. Frag For	GGGGCATTTCCCGTTCGTCATTAGCAGTAGGTAATGAAGATGC
	2. Frag Rev	CCCACAAATGTAGCTTCAAGCGCACGGATCGGAAGCGCCTCTG
	3. Frag For	CAGAGGCGCTTCCGATCCGTGCGCTTGAAGCTACATTTGTGGG
	3. Frag Rev	CCAAATGGGCAGGATCGGCCGCTCTAGTGATATCCTATTAGCG
Vash C-terminal endogenous tagging	Vash-C-tag for	TGACAACTTGCCAGGCAACTGACCGCTGGATGTTGCCAAATTCAGCAAAGGGTGCCAATATTAACGATGTGGGCCGATCGGGTTCTGGTAGTGGTTCC
	Vash-C-tag rev	ATTAAGAGGACATTCTACACGCACACCTCGCACACACACACACACACCCTTTGCCGCTGGGAGTGATCACAAACACAAAACCAATTTGAGAGACCTGTGC

Table S4. Reaction setup for the qPCR analysis.

Cycles	Name	Temperature	Duration
1x	Initial Denaturation	95°C	10 min
40x	Denaturation	95°C	15 s
	Annealing	55°C	30 s
	Extension	72°C	30 s
1x	Final Extension	72°C	5 min

Biology Open • Supplementary information
